# The current landscape of the antimicrobial peptide melittin and its therapeutic potential

**DOI:** 10.3389/fimmu.2024.1326033

**Published:** 2024-01-22

**Authors:** Hai-Qian Zhang, Chengbiao Sun, Na Xu, Wensen Liu

**Affiliations:** ^1^ Changchun Veterinary Research Institute, Chinese Academy of Agricultural Science, Changchun, Jilin, China; ^2^ Academic Affairs Office, Jilin Medical University, Jilin, Jilin, China

**Keywords:** melittin, pharmacological effect, nanomodification, immuno-conjugation, structural regulation, gene technology strategies

## Abstract

Melittin, a main component of bee venom, is a cationic amphiphilic peptide with a linear α-helix structure. It has been reported that melittin can exert pharmacological effects, such as antitumor, antiviral and anti-inflammatory effects *in vitro* and *in vivo*. In particular, melittin may be beneficial for the treatment of diseases for which no specific clinical therapeutic agents exist. Melittin can effectively enhance the therapeutic properties of some first-line drugs. Elucidating the mechanism underlying melittin-mediated biological function can provide valuable insights for the application of melittin in disease intervention. However, in melittin, the positively charged amino acids enables it to directly punching holes in cell membranes. The hemolysis in red cells and the cytotoxicity triggered by melittin limit its applications. Melittin-based nanomodification, immuno-conjugation, structural regulation and gene technology strategies have been demonstrated to enhance the specificity, reduce the cytotoxicity and limit the off-target cytolysis of melittin, which suggests the potential of melittin to be used clinically. This article summarizes research progress on antiviral, antitumor and anti-inflammatory properties of melittin, and discusses the strategies of melittin-modification for its future potential clinical applications in preventing drug resistance, enhancing the selectivity to target cells and alleviating cytotoxic effects to normal cells.

## Introduction

**Figure 1:**
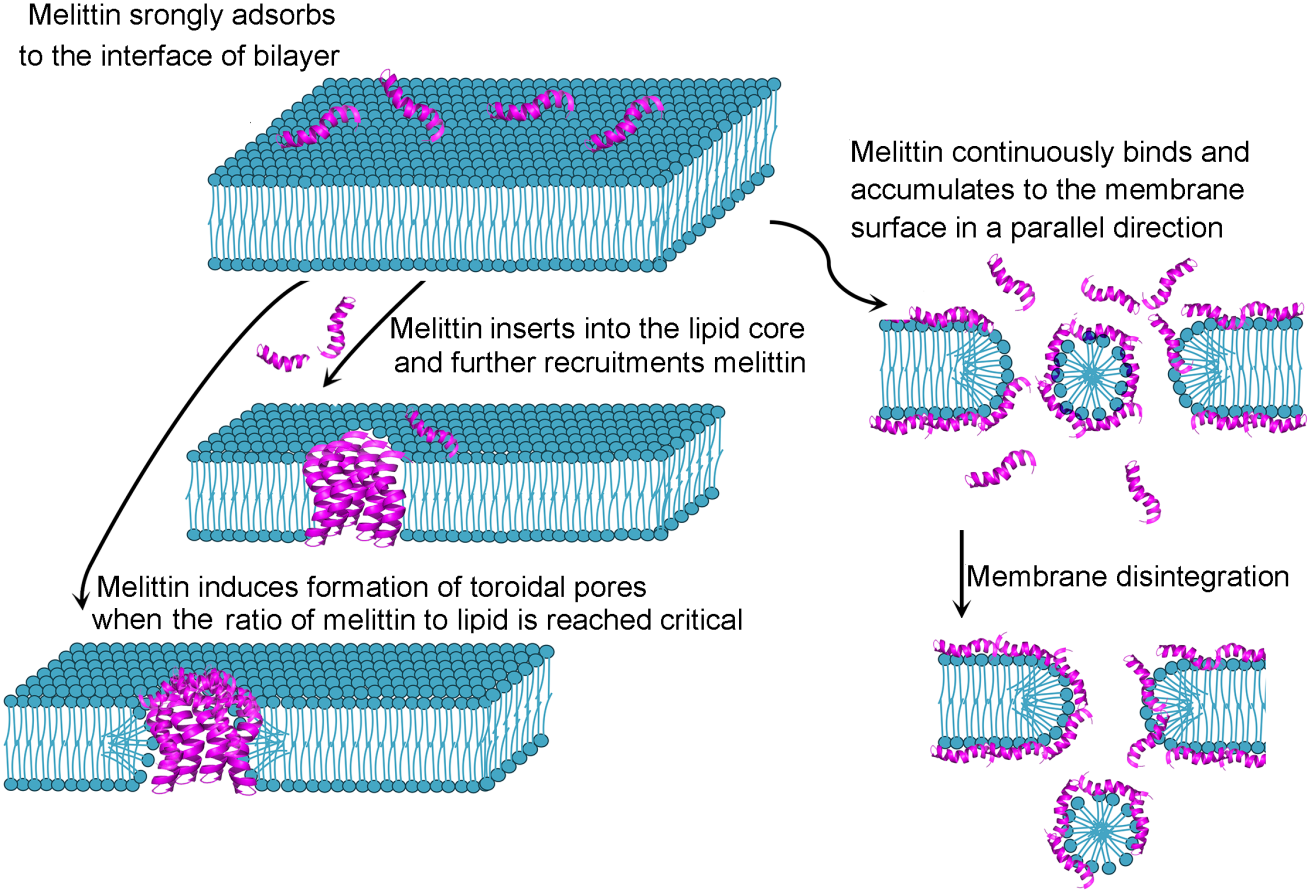
Possible model of melittin interacting with membrane.

Antimicrobial peptides have attracted increasing attention because of their potential benefits in agriculture, the food industry and medicine (1, 2). Antimicrobial peptides, naturally produced bioactive small proteins composed of a few amino acids by all living organisms, can protect against fungi, viruses and bacteria (2, 3). Melittin, the principal component of venom derived from honeybee *Apis mellifera*, is a promising antimicrobial peptide. Melittin is rich in *Apis mellifera* venom and can also be artificially synthesized. Melittin (C_131_H_229_N_39_O_31_) is a cationic short peptide weighting 2840 Da that consists of 26 amino acid residues (GIGAVLKVLTTGLPALISWIKRKRQQ-CONH2) (4) with 4 positive charges in the N-terminal portion and 2 positive charges in the C-terminal region (5). Due to the uneven distribution of polar and the nonpolar amino acid residues, melittin exhibits hydrophilic and lipophilic amphipathic characteristics. The amino terminal region (residues 1-20) plays a hydrophobic role, whereas the carboxyl terminal region performs a hydrophilic role because of a positively charged amino acid (6) in melittin. Each melittin chain is exhibited the structure of a helix-hinge-helix motif or bent rod. Amino acid residues 3-10 constitute an α-helix, and residues 12 to 14 form a hinge region, which is joined by an α-helix containing residues 15-24 (7, 8). Four-identical monomers of melittin compose a tetramer, which is the predominance in the abdominal sack of bee (7). When the venom is released, the tetramer is dissociated, yielding the monomer (9, 10). Melittin monomers can attach to the membrane surface, spontaneously bind to natural and artificial membranes (11) by inserting into phospholipid bilayers so that the strict of polar and non-polar moieties across the bilayer are weakened, and the permeability barrier is reduced. Based on the single-molecule dynamics perspective, Xu and colleagues (12) showed that the changes of membranes structure occurred when melittin was adsorbed and accumulated on the surface of cells membrane, which markedly accelerating the lateral diffusion of lipids around the melittin, accordingly, the diffusion facilitated melittin insert in such heterogeneous regions. The inserting process of melittin forms a “U”-bending pathway, takes shapes of toroidal pores with dynamical translocations of lipids and melittin in the lipid bilayer. Dye leakage and quartz crystal microbalance fingerprinting analysis showed that the disrupting action of melittin to membrane were differences response to different types of membrane: melittin-mediated surface-acting mechanism in lipid mixtures (bacterial-mimetic), penetrating the bilayer (mammalian-mimetic) and preferring for the domain containing predominantly zwitterionic lipids (domain-forming mixed membranes). It is proposed that melittin-induced membrane permeabilization may be due to toroidal pore or fissure formation in the membrane (13) (**Figure 1**). Melittin can be applied to monitor lipid-protein interactions in membranes (5, 14). Interestingly, electrochemiluminescence (ECL) imaging efficiently characterize the instant release of vesicle content via melittin-triggered permeabilization (15). Melittin is also applied for the studies related to calmodulin (CaM), a sensor protein of calcium ions, which is regarded as hub protein to bind and modulate more than 200 target proteins in a concentration-dependent manner of calcium ions. Recently, it is reported that the interaction of melittin and CaM is through melittin anchoring at the hydrophobic pockets of CaM by different sets of residues responding to different conformers (16). The multiple binding modes of melittin-CaM suggest the multiple target potential. Melittin can exert functions through nonmembrane-breaking pathways. A series of studies (17–20) on cardiovascular system diseases and neuropathic pain have reported that melittin exerts antiviral, anti-inflammatory and immune regulatory effects. Melittin could mitigate *Staphylococcus aureus*-triggered skin infection in CD1 mice and exerted protective effects in a model of methicillin-resistant *S. aureus* (21), which is significantly difficult to treat with available antibiotics (22). CD1 mice is widely used in the fields of pharmacology, toxicology and *etc.* because there are less differences in the growth and reproductive performance between different closed colonies raised in different areas of CD1 mice (23), which provides support for the applicable potential of melittin. Melittin inhibits the generation of inflammatory mediators and alleviates the inflammatory reaction in animal models with acute edema and chronic arthritis (24). It is worth noting that melittin can alleviate chemotherapy−induced peripheral neuropathy and ameliorate neuropathic pain in a rodent model (25). The Food and Drug Administration (FDA) approved melittin for relieving pain and swelling triggered by bursitis, tendinitis, rheumatoid arthritis and multiple sclerosis (26, 27). Melittin could hamper the proliferation and growth of human monocytic leukemia cells (28–30). Ceremuga and colleagues observed that melittin could induce the apoptosis of acute lymphoblastic leukemia and chronic myelogenous leukemia cells (31). Melittin suppresses the metastasis of hepatocellular carcinoma tumors (32), inhibits tumor growth in Colon26 NL-17-bearing mice *in vivo* (33) and exhibits synergistic effects with epirubicin in suppressing colon cancer *in vitro* (34). The clinically applicable potential of melittin and its functional mechanism are popular issues in the field of antimicrobial peptide research. Given that melittin possesses multi-target mechanisms of biological activity, melittin is considered an attractive candidate for therapeutic diseases in clinic. Therefore, this systematic review aims to summarize some of the important aspects related to melittin-mediated antiviral, tumoricidal and anti-inflammatory action. The article also discusses the different modification on melittin such as nanocarrier, immuno-conjugation, peptide-structural regulation and gene technology strategies for preventing the severe hemolytic activity and facilitating therapeutic targeting.

## Antiviral properties of melittin

Viral infections can be highly contagious and can cause serious harm to the body, which is reflected in the high fatality rate of many viral infections. Antiviral drugs mainly focus on disrupting the virus replication cycle. However, viruses proliferate quickly and are prone to mutation and drug resistance. The available drugs for targeted treatment are relatively limited. Seeking effective strategies to rapidly inhibit viral replication and prevent or delay drug resistance has attracted increasing attention in the exploration of antiviral agents.

A series of studies have shown the antiviral properties of melittin against herpes simplex virus (HSV) (35–39). HSV mainly attacks the skin, mucous membranes and nerve tissues. Human infection with HSV is relatively common. The sites of viral invasion and lesions vary. Neutralizing antibodies appear in the blood approximately one week after infection, reaching a peak in 3 to 4 weeks and lasting for many years. It is not clear if the protection of neutralization is sufficient although the vaccine elicited neutralizing titers and antibodies to HSV (40). One month after the final administration of HSV-2 gD2 vaccine, mean neutralizing titers against HSV were well-below than those of natural infection (41). Currently, there is no specific drug to cure HSV infections, and treatment strategies mainly include shortening the time of lesion healing or episode duration (42), preventing secondary infections and reducing recurrence. Baghian and colleagues have reported that melittin can impede cell fusion triggered by HSV-1 one of nine herpes viruses that infect humans. The researchers observed that melittin inhibited the attachment and penetration of HSV-1 to the membrane of host’s cell, which are major steps of replication cycle in infecting humans of viruses (43). The enveloped viruses attach to the plasma membranes and form close apposition and then release the viral nucleocapsid into cytosol via two-membrane fusion. Membrane microenvironment affects the entry of enveloped viruses (44). Melittin suppressed the activity of the Na^+^ and K^+^ pumps in infected cells (36), which may affect biological processes, such as metabolic reactions, transmembrane transport, adenosine triphosphate (ATP) synthesis and membrane fusion processes, indicating melittin-triggered inhibition of HSV-1 infection. Melittin suppresses HSV-1-triggered fusion of cells by suppressing HSV-1 glycoprotein K, which is necessary for efficiently encapsulating and translocating infectious virions from cytosol to extracellular fluid (45). Melittin exhibited virucidal activity when coincubated with HSV in African green monkey kidney-derived Vero cells (37), which was probably attributed to the direct interaction between melittin and the enveloped virus surface, destabilizing the membrane structure of the virus and inactivating the viral particles (38). Moreover, Albiol Matanic and colleagues showed that melittin impeded HSV 1 and HSV 2 infection but was highly toxic to host cells by osmotic lysis. Melittin can quickly integrate to lipid bilayers, oligomerize and form pores (39). Melittin can induce cell apoptosis (46) and necrosis (47, 48). Discovering strategies that reduce the toxicity of melittin is valuable and necessary for the feasibility of its clinical application.

Human immunodeficiency virus (HIV) leads to the severe impairment of immune function. Patients often suffer from severe opportunistic infections. HIV destroys the human antiviral defense system and promotes the development of cancer. The virus may carry oncogenes that can then be integrated into the DNA of host cells, causing the cells to undergo cancerous transformation, especially when the immune system is suppressed and immune surveillance is lost (49). Anti-HIV management is an important topic in the field of clinical treatment and medical research. It is worth noting that Gag, the structural protein of HIV-1, plays a decisive and specific role in the replication process of HIV-1 apart from being a scaffolding protein (50). Gag coordinates specific encapsulation of genomic RNA and motivates the formation of viral particles through its automatic assembly. Gag binds to a variety of viral proteins and interacts with intracellular proteins that are needed for its function and then translocates from its translation site to the plasma membrane, allowing the novel virions to be released. Therefore, Gag is considered a target for anti-HIV intervention. Wachinger and colleagues observed that melittin reduced HIV-1 production by affecting the processing of the Gag precursor protein (51). Moreover, melittin significantly reduced HIV-1 replication by damaging HIV proteins in a dose-dependent manner. Wachinger and colleagues obsderved that melittin suppressed viral replication in acutely HIV 1-infected human T lymphoma KE37/1 cells. Under melittin treatment, the levels of intracellular Gag and viral mRNAs were reduced, suggesting that melittin-mediated inhibition of HIV-1 replication occurred through interference with the host cell-directed gene expression of the virus (52). Considering the cytotoxicity of melittin, Hood and colleagues developed lipid film-based nanoparticles loaded with mellitin and investigated the effects of the nanoparticles on HIV-1 infectivity and biosafety. The researcher observed that melittin nanoparticles could capture more HIV-1 and significantly inhibit HIV-1 infection without presenting significant cytotoxicity (53). Interestingly, Jallouk and colleagues enveloped melittin into a lipid shell-perfluorocarbon nanoparticle to prevent its cytotoxic effects and enhance its therapeutic efficacy (54). The researchers showed that compared with free melittin, melittin-loaded nanoparticles reduced HIV infection and exhibited less toxicity on sperm and vaginal epithelial cells.

Severe acute respiratory syndrome coronavirus 2 (SARS-CoV-2) is a crown-like envelope RNA virus with a diameter of 120-160 nm. Patients suffering from SARS-CoV-2 exhibits clinical symptoms, such as ever, cough and fatigue, and showing ground-glass lesions in both lungs determined by Computed Tomography imaging. A few patients with severe SARS-CoV-2 infections present cytokine storm syndrome and hyperinflammation. Some cases even develop metabolic acidosis, coagulopathy and multiorgan failure (55, 56). Based on virus biology and pathology, researchers have proposed antiviral agents and immunomodulatory drugs to mitigate the severity of SARS-CoV-2 infection. The SARS-CoV-2 spike protein directly interacts with angiotensin-converting enzyme 2 (ACE-2) receptors in the body, initiating viral replication (57, 58). it is considered a strategy to disrupt the entry of the virus into cells and propagation by preventing the binding of the spike protein with ACE2. Enayathullah and colleagues observed that mellitin treatment could protect Vero cells against SARS-CoV-2 infection (59). The levels of the receptor binding domain (RBD) of the SARS-CoV-2 spike protein were reduced under melatonin treatment in SARS-CoV-2-infected Vero cells. Interestingly, molecular docking assays showed that melittin can interact with the RBD of the SARS-CoV-2 spike protein, suggesting melittin can reduce spike protein-ACE2 interactions. Moreover, the main protease Mpro (3CLpro) performs an important function in SARS-CoV-2 virus replication. Coronaviruses hijack the translational machinery of the host, generating viral proteins. The viral proteins need to be cleaved so that the individual functional proteins can be released for viral replication and transcription. Mpro cleaved viral proteins, facilitating the assembly of viral replicase complex (60). Mpro is considered a viable target for anti-SARS-CoV-2 virus intervention (61). Al-Rabia and colleagues developed a complex using sitagliptin (SIT) and melittin (MEL) to investigate the antiviral effects (62). The researchers demonstrated that the cellular uptake of SIT-MEL was significant in Vero E6 cells infected by SARS-CoV-2 virus. Moreover, Mpro-mediated inhibition was markedly higher with SIT-MEL complex treatment than under SIT or MEL treatment conditions. However, antimicrobial peptides have other limitations in addition to cytotoxicity, such as being easily hydrolyzed and eliminated from the bloodstream or causing allergic responses (63).

The anti-influenza A efficacy of melittin have been reported (37). Uddin and colleagues showed that melittin could markdely suppress the replication of influenza A virus in Madin-Daby canine kidney cells *in vitro*. Additionally, melittin treatment also exhibited protective effects against influenza A virus subtype H1N1-infected C57BL/6 mice *in vivo*. The mice suffering from H1N1 infection presented clinical signs of respiratory disease, such as ruffled fur, decreased activity, hunched posture and huddling, whereas melittin-treated mice did not show the above signs. Moreover, mice in the melittin treatment group showed lower viral titers in the lung than H1N1-infected mice without melittin treatment. Compared with HSV1 and HSV2, Junin virus (JV) is more sensitive to the inhibitory effects of melittin (64). JV is the etiologic agent of Argentine hemorrhagic fever (AHF). *Calomys musculinus* is the natural host of JV. JV can be transmitted to humans via the inhalation of aerosolized secretions and excretions of rodents (65). The onset of AHF is slow, and there are no specific symptoms during the early onset of AHF, which may delay the diagnosis. Accompanied by the debilitating hemorrhagic phase, the mortality rate of untreated AHF is 20% to 30% (66). Argentine researchers have been working to identify an effective treatment for AHF. Albiol Matanic and colleagues (64) showed that at a noncytotoxic concentration of 3 µM, melittin could reduce the virus infectivity of HSV-1, HSV-2 and JV by 80%, 80% and 99%, respectively. Melittin also exerts antiviral properties against enterovirus 71 (EV-71) (37). EV-71 is one of the major causative agents that causes hand, foot and mouth disease in young children, which leads to neurological, cardiac and respiratory complications (67). Viral infection with EV-71 is a serious threat to children’s health. Melittin treatment could reduce EV-71 replication and ameliorate EV-71 infection-mediated cytopathic effects in HeLa cells *in vitro*. The antiviral activity of melittin against several kinds of virus associated with human diseases are shown in **Supplementary Table 1**.

## Antitumor potential of melittin

Cancer is a global health problem and is one of the leading causes of death in the world. In recent years, substantial progress has been made in cancer diagnosis and treatment in recent years. However, traditional anticancer treatments still has severe side effects and toxicity and patients still exhibit drug resistance. It is crucial to identify new and effective anticancer treatments with low cytotoxicity. The low toxicity and tumor targeting abilities of antimicrobial peptides suggest their potential as antitumor agents. The antitumor effect of melittin is a popular topic (**Supplementary Table 2**). In addition to directly punching holes in tumor cell membranes, melittin can modulate the cell cycle, proliferation, angiogenesis metastasis and apoptosis of cancer cells (68).

Colon cancer is the second leading cause of cancer-related deaths worldwide (69). Main strategies for colon cancer treatment are surgery and chemotherapy. The critical problems of chemotherapy are cytotoxicity, nonspecific targeting, and drug resistance. Yaacoub and colleague evaluated the anticancer effects of melittin on a human colon carcinoma HCT116 cells (70). They observed that melittin had inhibitory efficacy on HCT116 cells in a dose-dependent manner, and there were significant synergistic effects between melittin and another main biopeptide of bee venom, phospholipase A2 (PLA2), on the inhibition of HCT116 cells. They speculated that melittin could destroy the glycocalyx of the cell coat on the cell membrane, which facilitated PLA2 directly acting on phospholipids of lipid membrane bilayer, probably causing the necrosis of HCT116 cells. Alfaleh and colleagues enveloped melittin with a fluvastatin nanoparticulate system containing phospholipid and alpha lipoic acid to assess the effects of the nanoparticles against colon cancer. The researchers reported that compared with monotherapy treatments, the hybrid system exhibited more significant inhibition of colon cancer Caco2 cell (71), and the hybrid nanosystem treatment arrested Caco2 cells in G2/M and pre-G1 phases (72). Additionally, melittin-mediated fast action on anticancer effects was reported in the cell lines of colorectal and gastric cancer *in vitro*. The researchers showed that melittin exhibited anti-tumor effects in a dose-dependent manner in the COLO205 cells (human colon cancer cell lines), HCT-15 cells (human colorectal adenocarcinoma cells). The morphological changes in above cell lines were evident within 30 seconds after melittin addition. Changes in the membrane were gradually observed, such as swelling, breakage or blebbing, and intracellular material was expelled from the cells (73). Melittin inhibited the growth and metastasis in MC38 cells (a murine colon cell line), and increased inflammatory cytokine levels in cancer cells (74). Luo and colleagues observed that melittin-mediated endoplasmic reticulum (ER) stress led to a disruption of calcium homeostasis and facilitated apoptosis in the human colorectal cancer cell line SW480 *in vitro*. Accordingly, the researchers showed that melittin management impeded tumor growth by triggering ER stress in SW480 tumor-bearing mice. No other marked biochemical and hematological toxicity to the mouse bodies were observed (75). The effects of melittin on the bone metastasis in colorectal cancer were assessed (76). A single dose of melittin was given to a mouse model of bone metastasis in human colorectal cancer (bearing HT-29 cells). Melittin treatment could inhibit the growth of metastases. Histological assays present necrosis and inflammatory morphology in melittin-treated metastases, suggesting the potential of melittin to reduce the growth of metastases in colorectal cancer patients.

Melittin treatment also impeded the growth of human gastric cancer (73). Melittin exhibited antitumor effects by inducing apoptosis in SGC-7901 cells (77). Melittin elevated the membrane permeability of mitochondria, reduced mitochondrial membrane potential and increased reactive oxygen species (ROS) release in SGC-7901 cells. Accordingly, melittin-induced increases in cytochrome C, apoptosis-inducing factor (AIF) and endonuclease G levels were consistent with the downregulation in second mitochondria-derived activator of caspases (Smac)/direct inhibitor of apoptosis gene (IAP) binding protein, activating caspase-3.

Drug resistance is still a critical problem for lung cancer therapy. Cellular mechanisms are related to resistance. Moreover, the tumor microenvironment (TME) is an important factor in the resistance of tumor cells to radiotherapy and chemotherapy (78). The TME is composed of immune cells and mesenchymal cells (79). A critical mechanism in the TME is the activation and infiltration of circulating monocytes, which differentiates into tumor-associated macrophages (TAMs). It is considered that TAMs may be involved in the regulation of tumor growth, infiltration and metastasis. Melittin arrested the cell cycle at G1 phase in a lung cancer cell line and induced apoptosis. Importantly, melittin could suppress the differentiation of circulating monocyte cells into TAMs (80), suggesting the antitumorigenic potential of melittin against lung cancer.

Melittin-mediated inhibition on the tumor invasion of breast cancer have been reported (81, 82). Breast cancer is the second most lethal cancer among women. The invasion and metastasis of cancer cells are the main causes of high mortality in patients suffered from breast cancer (83). The treatment strategies for breast cancer mainly include immunotherapy that targeting breast cancer-related genes, namely, nonspecific expression of human epidermal growth factor receptor (HER2), estrogen, or progesterone receptor and chemotherapy for triple-negative breast cancers (TNBCs). Chemotherapy causes damage to the whole body. Furthermore, once breast cancer resistance to one chemotherapeutic drug develops, it may also promote resistance to other chemotherapeutic drugs, that is, multidrug resistance, thus limiting treatment options. Seeking effective agents to alleviate side effects and overcome acquired resistance to chemotherapeutic drugs in breast cancer is a serious problem that needs to be solved. Considering that approximately fifty percent of TNBC patients overexpress epidermal growth factor receptor (EGFR), the activation of receptor tyrosine kinase (RTK) confers oncogenic regulation via the phosphatidylinositol 3-kinase/protein kinase B (PI3K/Akt) signaling pathway and HER2-enriched breast cancer patients specifically expresses HER2 (84), targeting EGFR may be a viable strategy for anti-breast cancer therapy. The selectivity of melittin to malignant breast cancer cells has attracted much attention (85). The tumoricidal properties of melittin on breast cancer cells have been investigated (86, 87). Melittin could impede primary tumor growth and inhibit the growth of the metastatic sentinel lymph node in breast cancer models *in vitro* and *in vivo* (88). Melittin directly acts on MCF7 breast cancer cells (81, 89) and suppresses the migration of breast cancer cell, MDA-MB-231 cell line (90). Melittin can effectively reduce the viability of human SUM159 cells (TNBC cell lines) (85). Scanning electron microscopy showed melittin-mediated membrane disruption of SUM159 cells. The levels of the apoptosis-related proteins, cleaved caspase-3, were markdely upregulated under melittin treatment. Moghaddam and colleagues showed that melittin-triggered apoptosis was associated with increases in the levels of mitochondrial fusion protein 2 and dynamin-related protein 1 in 4T1 TNBC cells (91). Interestingly, Duffy and colleagues designed a bifunctional melittin peptide in which an alpha-helical RGD peptide motif containing arginine, glycine and aspartic acid was added to the N-terminus of melittin. RGD-mellitin enhanced the targeting of melittin to the cell membranes in human TNBCs and T11 cells through a charged sequence present in the C-terminus of melittin. Melittin treatment downregulated the protein expression of phosphorylated RTK and the PI3K/Akt signaling pathway (85). Importantly, researchers have demonstrated synergistic effects between melittin and the chemotherapeutic agents docetaxel (85) and epirubicin (34). The resistant of tumor to docetaxel could be effectively treated under melittin administration in T11 xenograft nude mice *in vivo*. Significant decreases of phosphorylated EGFR and HER2 were also observed in T11-cell-bearing mice treated with melittin. Mir Hassani and colleagues reported that melittin impeded the growth of MDA-MB-231 cells, which is related with melittin-caused suppression on the TME formation (92). Hypoxia-inducible Factor 1 (HIF-1) is one of major drivers for decreasing the pH value of the solid TME by anaerobic respiration, effecting the metabolism of tumor cells (93). The alpha subunit of HIF-1 (HIF-1α) determines the activity of HIF-1. Interestingly, melittin induced apoptosis of MDA-MB-231 cells. Melittin management reduced the protein expressions and the mRNA levels of HIF-1α in MDA-MB-231 cells, suggesting melittin-induced suppression of TME formation. Further research is needed to describe the detailed targeting of melittin on breast cancer cells and its mechanism as well as its effects on healthy cells.

Lv and colleagues showed that melittin could inhibit the proliferation, migration and invasion of human hepatocellular carcinoma (HCC) cell lines, MHCC97-H and HepG2 (94). HCC is now the 3^rd^ leading cause of tumor-related death. The increasing mortality rate of HCC is attributed to limited therapeutic agents and the lack of curative treatments (95). Comprehensive treatment is a strategy to improve the prognosis and survival quality of patients suffering from HCC. Sorafenib was an approved agent used for advanced HCC chemotherapy. However, Sorafenib has severe side effects and induces drug resistance, so safe substitutes or adjuvants against advanced HCC are urgently needed (96). Mansour and colleagues reported that melittin synergistically potentiates the virucidal activity of sorafenib in HepG2 cells (97). The combined treatments of melittin with sorafenib (Mel/Sorf) exhibited a synergistic and selective inhibitory effect on HepG2 cells relative to normal liver THLE-2 cells and showed a higher cytotoxic effect on HepG2 cells compared with single treatments. Mel/Sorf treatment could disrupt the cell cycle, arresting HepG2 cells at G2/M phase (97). Computational assays showed that melittin binds to X chromosome-linked inhibitor of apoptosis (XIAP). The elevation of XIAP expression levels was significant in HCC (98). Shi and colleagues observed that a large number of samples from patients with advanced HCC exhibited high expressions of XIAP protein (99). A markedly increased risk of relapse may occur in the patients with XIAP-positive HCC that underwent total resection and orthotopic transplantation of liver. Inhibitors of apoptotic proteins might be a potential anticancer strategy for hepatocellular carcinoma (100). Zhang and colleagues showed that melittin could inhibit cathepsin S-induced angiogenesis and strongly impeded proliferation, invasion and angiogenesis in the HCC cell line MHCC97-H (101). Cathepsin S is a member of the lysosomal cysteine protease family. Cathepsin S degrades many extracellular matrix (ECM) proteins, such as fibronectin, laminin, collagens and elastin (102), promoting TME formation-related immune suppression and tumor growth (103). Cathepsin S is significantly upregulated in the liver of patients with HCC (104). A series of studies (105, 106) showed that downregulating cathepsin S could inhibit the growth, invasion and angiogenesis of tumors. Interestingly, melittin specifically inhibited tumor cell growth and impeded the activity of cathepsin S in HCC cells and Mock/MHCC97-H cells. Moreover, under melittin treatment, the protein levels of RAS, vascular endothelial growth factor (VEGF)-A, phosphorylated VEGF receptor 2 (VEGFR-2), RAF and mitogen-activated protein kinase kinase 1 (MEK1)/extracellular signal-regulated kinase (ERK)1/2 were strongly reduced in Mock/MHCC97-H cells (101). Targeting inhibitors to regulate RAS-RAF-MEK-ERK pathway has attracted increasing attention in current research and treatment for cancer (107). Melittin-mediated effects on Mock/MHCC97-H cells show potential for HCC therapy, although more experiments are needed before clinical studies can be carried out. Zhang and colleagues also showed that melittin inhibited the proliferation of HCC and HepG2 cells by downregulating histone deacetylase 2 (HDAC2), leading to the upregulation of a tumor suppressor gene, phosphatase and tensin homolog deleted in chromosome ten (PTEN) (108). HDAC2 is associated with the DNA damage response (109). Shan and colleagues reported that increases in HDAC2 expression levels were significantly related to high-grade tumors, lymph node metastasis and poor prognosis (110). The upregulation of HDAC2 could prevent apoptosis in tumor cells (111). HDAC2 was considered a key target for anticancer treatment (112). The overexpression of HDAC2 was present in patients suffering from HCC (113). Melittin-triggered inhibition of HDAC2 suggested the potential of melittin as a treatment against HCC. Melittin can induce autophagy in HepG2 cells, activating the mitochondrial apoptotic pathway (114). Furthermore, melittin treatment downregulated DNA methyltransferase protein-1 (DNMT1). DNMT1 demethylated and upregulated long noncoding RNA (lncRNA) ADAMTS9-AS2 (94), which is a tumor suppressor (115). Melittin-mediated upregulation of ADAMTS9-AS2 provided new insights into the potential of melittin as a treatment against HCC. The anti-metastatic role of melittin was evaluated. Liu and colleagues demonstrated that melittin impeded the viability, microfilament depolymerization and migration of MHCC97L and MHCC97H HCC cells *in vitro*. Moreover, under melittin treatment, tumor volume was significantly reduced in nude mice bearing MHCC97H xenografts, whereas the body weight of the mice increased compared with that of tumor xenograft nude mice without melittin treatment. Interestingly, melittin triggered the inhibition of Rac1 (32), a member of the Rho GTPase family. Rac1 is considered to be involved in cancer cell metastasis (116). Several studies have explored the strategy of targeted modification of melittin to enhance its selective and antitumor properties against HCC. Melittin was linked to a homing peptide, SLSLITMLKISR (AM-2), using A(EAAAK)2A to analyze the virucidal activity of this ligation (117). After being incorporated into AM-2, the affinities of melittin to HepG2 cells were elevated, and cell growth was inhibited, suggesting that AM-2 ligand-mediated immunoconjugation may be a valuable strategy for HCC cell targeting. Zhao and colleagues constructed a recombinant immunotoxin by fusing an anti-asialoglycoprotein receptor (ASGPR) single-chain variable fragment antibody (C1) to melittin (C1M). They observed that the C1M could bind the surface of HepG2 cells and exhibit cytolytic capacity to HepG2 cells without causing erythrocyte dialysis (118). Qian and colleagues assessed the effects of melittin gene therapy. Melittin gene was carried by an adenovirus vector containing the hypoxia-response element (HRE)-alpha fetoprotein (AFP) promoter to control adenovirus E1a gene expression to target AFP-positive cancer cells under a hypoxic microenvironment. AFP-positive HCC cells were significantly inhibited under the above adenovirus carrying melittin, while under the targeted delivery of the above adenovirus, melittin management suppressed the growth of HCC cells and Hep3B cell xenografts, and increased the overall survival of nude mice bearing Hep3B cells in comparison with those of tumor-bearing mice without melittin treatment (119). Survivin is a member of the inhibitor of apoptosis family. Survivin is transcriptionally urpegulated in most malignant tumors, and its promoter is tumor specific. Using the survivin promoter (pSURV-Mel), Qu and colleagues developed a nonviral vector encoding the melittin gene to assess the anticancer effects of pSURV-Mel in HepG2 cells *in vitro* and a mouse model bearing human HepG2 cells *in vivo* (120). The results showed that pSURV-Mel was selectively expressed and induced cytotoxicity in human HepG2 cells but not in the normal liver cell Line L02. pSURV-Mel induced death of HepG2 cells through an apoptosis-dependent pathway. Ling and colleagues developed a recombinant adenovirus carrying AFP promoter and melittin gene (Ad-rAFP-Mel) to analyze the antitumor effects in human HCC cell line BEL-7402 *in vitro*. They showed that the tumorigenicity rate in BEL-7402 cells transfecting Ad-rAFP-Mel was significantly reduced. In addition, Ad-rAFP-Mel intratumoral injection exhibited marked antineoplastic effects on transplanted tumors in nude mice (121). Anti-HCC effects of Ad-rAFP-Mel were confirmed by Li and colleagues (122). Ad-rAFP-Mel treatment could inhibit cells proliferation and lead to chromatin condensation and nuclear fragmentation in BEL-7402 cells.

The effects of melittin on prostate cancer were evaluated in PC-3, LNCaP and DU145 cells. Melittin treatment could induce apoptotic cell death by inhibiting NF-κB and upregulating caspase signaling (123). Chlorotoxin (CTX) is regarded as one of cancer cell-targeting ligands due to its capacity to recognize different tumors (124). CTX could specifically interact with matrix metalloproteinase-2 (MMP-2) and impede MMP-2 activity, which may affect tumor metastasis process (125). Tarokh and colleagues constructed a CTX-targeted nanovector to deliver the melittin gene (126). The CTX-targeted nanovector selectively increased the transfection efficiency of melittin gene in MMP-2 positive human prostate cancer cell line PC3 and inhibited cell viability without affecting the growth of MMP-2 negative NIH3T3 cell line, suggesting the antitumor selectivity of melittin under CTX-targeted nanomodification. In addition, Yan and colleagues showed that melittin could enhance cisplatin sensitivity and inhibit migration of in castration-resistant prostate cancer cells and PC3 cells (127). Interestingly, melittin-triggered tumor impediment and cisplatin sensitivity could be abrogated by the overexpression of lipocalin-2 (LCN2), a gene of the IL-17 signaling pathway. It is nominated the potential of melittin as a candidate for CRPC therapy.

## Anti-inflammatory potential of melittin

Inflammation is a defense response of the body against injury. The body maintains homeostasis by limiting infection and repairing damage. However, chronic inflammation can trigger or exacerbate a variety of diseases, such as arthritis, heart disease, arteriosclerosis, inflammatory bowel disease, asthma, central nervous system disease and diabetes. It was reported that melittin could inhibit excessive inflammation, which suggests the melittin can treat inflammation-induced diseases (**Supplementary Table 3**).

Melittin effectively mitigates the lipopolysaccharide (LPS)-mediated pro-inflammatory response in mouse BV2 microglia (128). LPS is thought to be a key molecule in the endotoxic shock associated with gram-negative infection, which is released during cell division, death or an inflammatory response (129). Moon and colleagues showed that melittin inhibited LPS-primed of nitric oxide/inducible nitric oxide synthetase (NO/iNOS) expressions in a dose-dependent manner in BV2 microglia. Melittin treatment reduced the protein expressions of a major mediator of inflammation, prostaglandin E2 (PGE2), and inhibited LPS-primed activation of nuclear factor-κB (NF-κB). Additionally, melittin inhibited the transcription of interleukin-6 (IL-6), interleukin-1β (IL-1β) and tumor necrosis factor-α (TNF-α) triggered by LPS in BV2 cells. Han and colleague analyzed the effects of melittin on free radical-triggered neuronal damage (130). H_2_O_2_ treatment was used to mimic the stimuli of free radicals in the human neuroblastoma SH-SY5Y cell line. Under chronic oxidative stress, cell viability was inhibited, and the protein expressions of Caspase-3 were increased, whereas melittin management significantly reduced DNA fragmentation and increased cell viability. Melittin inhibited H_2_O_2_-triggered downregulation of BCL-2 in the protein expressions and mRNA levels. In addition, melittin ameliorated H_2_O_2_-induced upregulation of pro-apoptotic factors Bax and Caspase-3 in the protein expressions and mRNA levels, indicating melittin-mediated neuroprotective effects against oxidative stress by inhibiting apoptosis in neuronal cells. Considering that oxidative stress is an important inducer of neurodegeneration, these results suggest the potential of melittin for neurodegenerative disease treatment. Amyotrophic lateral sclerosis (ALS) is associated with mutations in superoxide dismutase (SOD1) in motor neurons, excitotoxicity, defective axonal transport, oxidative damage, mitochondrial dysfunction, protein misfolding, abnormal cytoskeleton and neuroinflammation. To analyze the effects of melittin on motor neurons of ALS-related pathological changes, melittin was administered to HSOD1G93A mice, an ALS model animal. Yan and colleagues showed that melittin treatment improved motor function in mice and alleviated neuronal damage in the spinal cord of HSOD1G93A mice. Melittin administration mitigated α-synuclein misfolding in the brainstem and spinal cord of HSOD1G93A mice, restoring the activity of proteasome in the brainstem and spinal cord (131).

Several researchers have investigated the potential of melittin against rheumatoid arthritis (RA). RA-caused irreversible destruction and functional loss of joints disturb the life of patients. Fibroblast-like synoviocytes (FLS) produce cytokines in the synovial intimal lining, perpetuating inflammation and proteases and then promoting cartilage destruction. Nah evaluated the effects of melittin on FLSs from patients with RA. Under LPS stimuli, the characteristic mediums mediating RA and matrix metalloproteinases (132) were significantly elevated. Under melittin treatment, LPS-induced increases in MMP-3 production and DNA binding activity of NF-κB were alleviated in FLS (133). Melittin-mediated inhibition of FLS was confirmed by He and colleagues (134). The researchers observed that melittin markedly inhibited the viability of FLS from patients with RA (RA-FLS), reduced the secretion of IL-1β and promoted apoptosis and autophagy in RA-FLS. FLS survival might depend on the activation of transcription factors, signal transducer and activators of transcription 3 (STAT3), which is regulated by IL-6 (135). It has been reported that inhibiting IL-6 (136) or the IL-6 receptor (137) could improve RA patients’ signs and symptoms. The cooperation of IL-6 and soluble IL-6 receptor (sIL6R) can induce FLS proliferation (138). Kim and colleagues showed that IL-6/sIL-6R stimuli activate the STAT3 and nuclear NF-κB p65 in FLS. Melittin treatment ameliorated IL-6/sIL-6R-triggered activation of STAT3 and nuclear translocation of NF-κB p65, increased the protein levels of pro-apoptosis-related proteins, apoptotic protease activating factor 1 (Apaf-1), caspase-9 and caspase-3, and increased the release of cytosolic cytochrome c (139).. Mitogen-activated protein kinase (MAPK) and NF-κB are abnormally activated in the inflammatory pathological process of RA, and the transmission of inflammatory signals among abnormal signaling pathways promotes the release of inflammatory factors (140). Melittin can interact with the P50 subunit (24) and thiol group of IκB kinase (IKK) (141) so that the IκB kinase loses its catalytic effect on the phosphorylation of IκB (inhibitor of NF-κB). NF-κB cannot enter the nucleus. P50 nuclear translocation is also inhibited, which inhibits the production of inflammatory mediators (141, 142). Park and colleagues confirmed that melittin treatment prevented LPS-induced transcription of NF-κB and impeded NF-κB DNA binding activity of by suppressing IκB release and p50 translocation (24). Interestingly, melittin treatment could reverse the LPS-primed activation of JNK and NF-κB in Raw 264.7 cells (a murine macrophage line), and the effects above were abolished by a JNK inhibitor (143), suggesting that the properties of melittin on anti-inflammatory and anti-arthritis were involved in regulating JNK signaling pathways.

Ulcerative colitis (UC) is a type of bowel disease with the characteristics of primarily affecting the superficial layers of the rectum and colon and lifelong inflammation (144). Routine therapies cannot achieve satisfactory efficacy, and the side effects cannot be ignored. More effective therapeutic agents are still needed for UC. Ahmedy and colleagues assessed the antiulcerogenic effect of melittin by oral administration in a colitis mouse model induced by acetic acid (145). Melittin treatment ameliorated acetic acid-triggered decreases of body weight and an increase of colon mass index in colitis model mice. The levels of PGE2, NF-κB, Toll-like receptor 4 (TLR4), tumor necrosis factor (TNF-α), IL-6, secretory phospholipase A2 (sPLA2), p38 MAPK, tumor necrosis factor receptor (TNF-R)-associated factor (TRAF6) and cyclooxygenase 2 (COX-2) were significantly reduced under melittin administration compared with those in colitis model mice without melittin treatment. In addition, melittin attenuated oxidative stress in colitis model mice. Anti-inflammatory property of melittin against UC was assessed in dextran sulfate sodium (DSS)-induced colitis models (146). The synergies between melittin and sulfasalazine were also analyzed to evaluate whether combination therapy could improve the clinical symptoms of UC model mice. The results showed that melittin and the combination of melittin with sulfasalazine significantly alleviated pathological damage in the colon tissue of UC mouse model. Moreover, melittin treatment exerted antioxidant effects on colitis model mice, suggesting that melittin might be a potential alternative agent for UC intervention.

Acute liver failure (ALF) occurs shortly after liver damage in people who have not previously suffered from liver disease. ALF is a severe clinical syndrome with extremely poor prognosis and high mortality. There is no curative treatment for ALF. It is considered that the high mortality rate of ALF may be due to the severe disorder of the systemic inflammatory response (147). Modulating macrophages to suppress inflammation is considered a promising strategy for ALF intervention (148). Park and colleagues assessed the effects of melittin against ALF in a mouse model caused by d-galactosamine (d-GalN) and LPS (d-GalN/LPS) intraperitoneal challenge *in vivo* (149). They showed that melittin attenuated the d-GalN/LPS-induced increase of IL-1β and TNF-α in the serum and liver of ALF model mice. Apoptosis was less in the melittin-treated d-GalN/LPS mice than those of d-GalN/LPS-treated group without melittin administration. Moreover, melittin management mitigated the activation of NF-κB in ALF model mice. Melittin-mediated protection against ALF failure by inhibiting inflammatory cytokine release was confirmed by Fan and colleagues (150). The researchers demonstrated the anti-inflammatory activities of melittin in ALF mouse induced by d-GalN/LPS *in vivo* and LPS-stimulated RAW264.7 macrophages *in vitro*. Researchers observed that LPS stimulation facilitated aerobic glycolysis in RAW264.7 cells by increasing the levels of Warburg effect-associated enzymes and metabolites, upregulating glycolytic rate, and enhancing Akt/mTOR/PKM2/HIF-1α signaling. The mortality of ALF mice were significantly reduced under melittin treatment, and the symptoms and signs of ALF were mitigated. Melittin management ameliorated inflammation and exerted antioxidative effects on RAW264.7 cells. Furthermore, melittin treatment attenuated LPS-primed increases in HIF-1α and PKM2 levels by alleviating LPS-triggered upregulation of Akt/mTOR signals in RAW264.7 cells and ALF model mice, and the melittin-mediated effects above were abolished by shRNA against PKM2. The authors considered that melittin alleviated inflammation in ALF through inhibiting PKM2-mediated Warburg effects.

Acne is a chronic inflammation of the sebaceous glands in the skin follicles, which is closely associated with the inflammatory reaction caused by *Propionibacterium acnes* (PAC). Lee and colleagues examined the activity of melittin against PAC-triggered inflammatory responses in THP-1 monocytic cells (151). They observed that melittin treatment attenuated heat-killed PAC-mediated increases of cleavage of caspase-3 and -8 and pro-inflammatory cytokines, and mitigated PAC-triggered apoptosis in THP-1 cells. In addition, melittin treatment alleviated heat-killed PAC-induced increases in proinflammatory cytokine levels and Toll-like receptor 2 expression levels by inhibiting MAPK and NF-κB signaling pathways in a human keratinocyte HaCaT cells. Melittin also attenuated swelling, cutaneous erythema and the granulomatous response in the ears of living PAC-injected mice than those of mice injected with living PAC without melittin treatment (152).

Melittin could exert anti-inflammatory effects against a kind of inflammatory skin disease, atopic dermatitis (also named atopic eczema). The main clinical manifestations of atopic dermatitis include epidermal thickening, skin dryness and pruritus, a decrease in filaggrin, damage to the epidermal barrier, various allergic reactions mediated by IgE, and disturbance of the immune system (153, 154). Kim and colleagues showed that melittin mitigated ovalbumin-triggered skin symptoms in an atopic dermatitis model mouse *in vivo*. Melittin treatment regulated skin thickness, increased filaggrin levels, reduced inflammatory cytokine and chemokine levels and alleviated the IgE response. Melittin could inhibit the phosphorylation of STAT3, reduce the secretion of IL-4 and IL-13 and increase the protein expressions of filaggrin in HaCaT cells. Further studies may provide more clues regarding melittin in atopic dermatitis treatment (155).

Periodontitis is a type of inflammatory disease affecting gums, periodontal ligament, alveolar bone and cementum, resulting in the destruction of the gingiva. *Porphyromonas gingivalis* (*P. gingivalis*) is a main pathogen of chronic periodontitis and can cause alveolar bone absorption and tooth loss by invading periodontal tissues and releasing toxic substances (156). Kim and colleagues evaluated the effects of melittin on *P. gingivalis* LPS (PgLPS)-infected HaCaT cells (157). They observed that melittin treatment ameliorated PgLPS-induced increases in the protein expressions of IL-6, IL-8, TLR-4, TNF-α and interferon-γ. Melittin incubation mitigated PgLPS-triggered activation of extracellular signal-regulated kinase, NF-κB and PKB/Akt.

## Discussion

### Melittin-induced cytotoxicity limits its biological applications

Melittin is characterized by antiviral activity; however, melittin-induced destruction of the membrane of red blood cells should not be ignored (4, 158, 159). Gajski and colleagues showed that there are morphological changes in the cell shape, membrane damage, cell granulation and lysis of human peripheral blood lymphocytes (HPBLs), apart from a decline in viability when exposed to melittin (158). Under melittin treatment, the levels of ROS were significantly increased, and oxidation-reduction homeostasis was disrupted. Melittin increased the formation of micronuclei, nuclear buds and nucleoplasmic bridges, suggesting that genome instability was triggered by melittin. Melittin treatment increases the mRNA levels of oxidative stress- and apoptosis-related genes, which suggests that it exerts cytotoxic effects on normal human cells (160). Melittin can cause tonic pain and peripherally persistent pain. Local injection with melittin could cause hyperalgesia, allodynia and inflammatory responses in the injection site (161). Melittin enhances the excitability of spinal nociceptive neurons. Local injection of melittin also leads to hyperresponsiveness of spinal wide dynamic-range neurons under mechanical and thermal stimuli, causing spontaneous nociceptive flinches and pain hypersensitivity (161). Melittin treatment by intradermal injection of triggered mechanical hyperalgesia and heat in the zone of primary injury in humans (162, 163). Subcutaneous injection of melittin triggers heat hypersensitivity by activating tetrodotoxin-resistant sodium channels in small primary sensory neurons (164). Melittin-caused toxicity to cells or to the host animal is not uniformly (165). The median hemolytic concentration (HC_50_) of melittin against human red blood cells (2% suspension) is 16.28 ± 0.17 µg/mL and the median lethal concentration (LC_50_) of melittin against immature human dendritic cells (1 × 10^5^/mL) is 43.42 ± 0.86 µg/mL when incubated for 24 h (166). Melittin can trigger morphological changes in mouse peritoneal macrophages at the concentration of 2.5 μg/mL, and the half-maximal inhibition (IC_50_) is 5.73 μg/mL (167). It is necessary to seek strategies to reduce the cytotoxicity and enhance the bioavailability of melittin.

### Packaging melittin by nanocarriers is one strategy to minimize the toxicity of melittin

Ligands such as oligonucleotide or peptide molecules can specifically bind to melittin with high affinity. In addition, researchers have shown that the synergistic effects of melittin with some first-line drugs could effectively reduce melittin cytotoxicity and drug resistance. Gui and colleagues developed a nanocomplex system based on polyelectrolytes to allow strong interactions formation between positively charged melittin and negatively charged dextran sulfate using flash nanocomplexation technology (168). They obtained uniform nanocomplexes with high melittin encapsulation efficiency, high payload capacity and tunable release profiles. When treated with free melittin by intraperitoneal (i.p.) injection, there was high acute liver toxicity and a low survival rate in a type II diabetes (T2D) mouse model. In contrast, there was no death in the mice treated with melittin-loaded nanocomplexes. The time of melittin release was prolonged. The blood glucose levels declined after melittin nanocomplex *i.p.* injection. Moreover, mouse body weight was reduced. The author considered that their melittin nanocomplexes reduced acute toxicity and improved pathological indicators, enhancing the applicable potential of melittin. Ye and colleagues (169) constructed lipid-coated polymer nanoparticles loaded with melittin, which exhibited suitable biocompatibility and stability. The nanosystem contained a PEG-targeting molecular shells and lipid membrane intermediate layer. The formulation was prepared by self-assembly according to intermolecular interactions, comprising hydrophobic effects and electrostatic attraction. Under physiological conditions, the melittin-loaded system exhibited a high encapsulation efficiency and stability. Hemolysis showed that free melittin induced lysis rate of red blood cells was100%. In contrast, neither remarkable hemolysis in the red cells nor specific cytotoxicity was observed under melittin-loaded nanosystem treatment, even at high concentrations of melittin. Nanomodification enhanced the selectivity of melittin for tumor cells and markedly inhibited the tumor growth without causing significant normal tissue toxicity or hemolysis in nude mice bearing the human lung adenocarcinoma xenografts.

Li and colleagues (170) designed liposomes with dioleoyl phosphoethanolamine (DOPE)-conjugated hyaluronic acid (HA) inserted by covalent bonds (HA-DOPE). Then, HA-modified liposomes were used to carry melittin (Mel-HA-Lip). HA and its derivatives can interact with specific receptors, such as HA receptor for endocytosis (HARE), receptor of HA-mediated mot and CD44 in the cell membrane (171). CD44 is a widely distributed glycoprotein. Overexpression of CD44 can be observed in the cell membrane of some malignant tumor cells. The specific binding of CD44 with HA can mediate transmembrane transportation (172, 173). The nature of specific binding between HA/HA derivatives and CD44 provides targeted delivery for antitumor drugs to tumor cells (174). HA-modified liposomes enhanced the uptake of melittin by B16F10 cells. Mel-HA-Lip exhibited higher levels of sustained-releases than Mel-Lip. Furthermore, Mel-HA-Lip performed more obvious antitumor effects compared with Mel-Lip (170). Dai and colleagues developed dual-targeting nanoparticles carrying melittin with HA-conjugated high-density lipoprotein (HDL) as the phospholipid scaffold (MLT-HA-HPPS) to target breast cancer and its sentinel lymph node (SLN) (88). MLT-HA-HPPS exhibited effective cytotoxicity on mouse mammary adenocarcinoma 4T1 cells. MLT-HA-HPPS paracancerous administration significantly suppressed the growth of primary tumor. It is noted that MLT-HA-HPPS could target SLN and impede the growth of metastatic SLN in the lymph node metastasis model bearing 4T1 cells. Tang and colleagues developed TME-responsive MnO2-melittin nanoparticles (M-M NPs) based on an *in situ* redox reaction between poly(allylamine hydrochloride) and KMnO4 (74). Melittin was carried by carbodiimide method. M-M NPs consumed GSH at a faster rate and facilitated more cytotoxic ROS (•OH) production, inducing tumor cell death without affecting the viability of antigen presenting cells, murine DC cells and bone marrow (BM)-derived macrophages. M-M NPs inhibited tumor growth and metastasis, and increased inflammatory levels in MB49 prostate cancer subcutaneous tumor model mice *in vivo* and in MC38 colon cancer and B16 melanoma *in vitro*. Importantly, M-M NPs facilitated MHC-I cross-reactivity via DC, activating tumor-specific CD8 positive T cells, which suggested the antitumor immunotherapy potential of MnO2-melittin.

### The peptide-peptide and peptide-drug conjugate strategy may increase the bioavailability and relative safety of melittin

A conjugated peptide-peptide fused with melittin (melittin 8-26) and the pro-apoptotic peptide d(KLAKLAK)2 (dKLA) could inhibit tumor growth and reduce mortality in a mouse model bearing 4T1 TNBC *in vivo* (82). Melittin-dKLA 8-26 treatment reduces the numbers of M2-like tumor-associated macrophages, which are the main components of TME and involved in the regulation of tumor invasion, angiogenesis, growth, migration and metastasis (71, 175). Additionally, under melittin-dKLA 8-26 administration, lung metastasis was significantly suppressed in 4T1 TNBC mice. Melittin could also increase ROS levels, triggering immunogenic cell death (176). Lee and colleagues (177) demonstrated that melittin-dKLA selectively inhibited M2-like TAMs in cancer mouse model bearing murine Lewis lung carcinoma cells *in vivo.* Melittin-dKLA could suppress the growth and lower the weights of tumor, and impeded angiogenesis. Jeong and colleagues (178) also showed that the peptide-drug conjugate derived by melittin could inhibit melanoma by targeting M2-like TAMs in a melanoma mouse model bearing B16F10 cells. The research conjugated melittin and mertansine (DM1), an inhibitor targeting the assembly of tubulin into microtubules and cell cycle. Melittin-DM1 significantly increased the survival rates of melanoma mice than those under DM1 management alone. Moreover, a series of studies (86, 87, 179) investigated several strategies, such as innocuous nonspecific toxicity and increased stability of melittin in anti-breast cancer therapy. Daniluk and colleagues showed that compared to melittin alone, using diamond, pristine graphene, graphene oxide and carbon nanoparticles as the carriers for melittin reduced the level of necrosis in MDA-MD-231 and MCF-7 cells (86). Through caveolin-dependent endocytosis, graphene-carried melittin can be transported to MDA-MD-231 cells (87). Melittin-graphene oxide complex reached MDA-MB-231 cells through phagocytosis, microtubule-dependent transport and caveolin-dependent endocytosis. Raveendran and colleagues (179) developed a polyion complex micelle modified by estrone for delivering melittin to hormone-responsive breast cancer cells.

Nabizadeh and colleagues (180) designed a hybrid peptide by fusing truncated-melittin (11 amino acids with substituting arginine for isoleucine in the 5^th^ position) to Lasioglossin (LL-III, 15 amino acids), which can suppress *Acinetobacter (A.) baumanni*. The hybrid peptide above was named LM1 and was demonstrated to be high stability. The interactions of LM1 with lipid membrane were increased compared with melittin and exhibited antimicrobial potential to halter *S. aureus bacteria* and *A. baumanii*. Huang and colleagues designed α-melittin, a hybrid cytolytic peptide, by linking an amphipathic α-helical peptide to the N-terminus of melittin using a GSG linker (181). α-Melittin can bind phospholipids and self-assemble, to form lipid nanoparticles. The lipid nanoparticle-carried α-melittin (α-melittin-NPs) shielded the positive charge of melittin, reducing its cytotoxicity. In addition, α-melittin-NPs treatment by intravenous injection suppressed the growth of tumor in melanoma-bearing nude mice. Interestingly, α-melittin-NPs could trigger a systemic antitumor response (182). In comparison with free melittin, α-melittin-NPs can facilitate the lymph node uptake of melittin, stimulating activation of antigen-presenting cells activation in C57BL/6 mice. Moreover, α-melittin-NPs could impede primary and distant tumor growth in a model of bilateral flank melanoma by intradermally implanted B16F10 cells. α-Melittin-NPs could also control T-cell-mediated chronic allergic contact dermatitis and atopic dermatitis skin (183). In an allergic contact dermatitis mouse model induced by oxazolone challenge in the ear, both melittin and α-melittin-NPs intradermal injection significantly alleviated ear swelling. In comparison with direct intradermal injection of melittin, α-melittin-NPs treatment alleviated the excess accumulation of leukocytes at the injection position without damaging the epidermal structure integration. α‐Melittin‐NPs target lymph nodes and suppress allergen‐induced maturation of dendritic cells in allergic contact dermatitis mice, whereas α‐Melittin‐NPs reduce the levels of phosphorylated-RelB (pRelB) and impede the activation and proliferation of CD8^+^ T cells. RelB is a critical signaling molecule of NF-κB signaling. Phosphorylation-activated RelB migrates to the nucleus, priming gene transcription associated with the maturation of dendritic cells (184). Interestingly, α‐melittin‐NPs controlled the release of Th1-type cytokines in allergic contact dermatitis mice and restricted the release of Th2-type cytokines and IgE in mice with atopic dermatitis-like pathology, indicating the immunosuppression against T cells-mediated immune reactions.

### Melittin might be used in synergistic therapeutics for medicine in clinic

**Figure 2:**
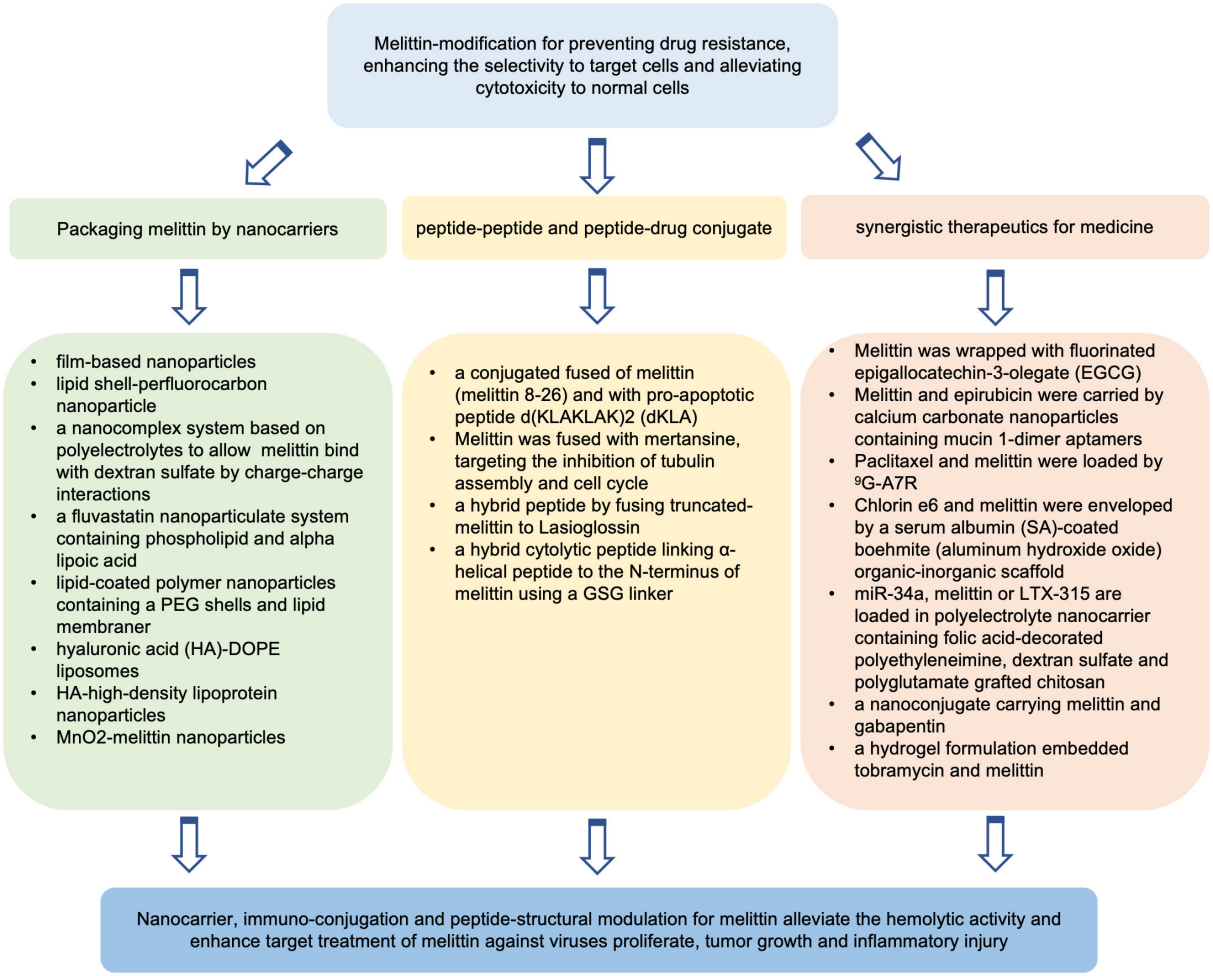
Potential strategies of melittin modulation for increasing its biosafety and therapeutic index.

Codelivery of melittin with antibiotics, chemotherapeutics, small molecule compounds, proteins or gene agents for synergistic therapeutics was explored to develop safer and more efficient strategies against disease. Through hydrogen-bond interactions, melittin (MPI) was wrapped with fluorinated epigallocatechin-3-olegate (EGCG) (185). The fluorination of EGCG occurred through the reaction of EGCG and fluorinated compounds by ROS-sensitive linker of oxalyl chloride. EGCG, a potential inhibitor of programmed death-ligand 1 (PD-L1), could regulate the response of tumor cells to immunotherapy (186, 187). The self-assembly of melittin and fluorinated EGCG (FEGCG) was formulated into a fluoro-nanoparticle system, FEGCG@MPI NPs, which may deliver small molecule drugs but also larger macromolecules (185). Fluorine modification of EGCG ameliorated the hemolysis induced by melittin and promoted the cellular uptake of FEGCG@MPI NPs by Hep3B cells. FEGCG@MPI NPs markedly reduced PD-L1 protein expressions of and disrupted the interactions of PD-L1 and programmed death-1 (PD-1). FEGCG@MPI NP treatment inhibited tumor growth and induced tumor cell apoptosis in HCC mice bearing Hep3B tumor cells. Yazdian-Robati and colleagues developed calcium carbonate nanoparticles (CCNs) with mucin 1-dimer aptamers to carry epirubicin and melittin by using a water-in-oil emulsion method (34). They showed that the codelivery of epirubicin and melittin by using a mixture of mucin 1-dimer aptamer-CCNs-melittin complex and micin 1-dimer aptamer-CCNs-epirubicin complex exerted significant synergistic cytotoxicity in murine colon cancer C26 cell line and MCF-7 cells. Furthermore, under the delivery of mucin-1-dimer aptamer-modified CNN, epirubicin and a mixture containing epirubicin and melittin exhibited more effective antitumor effects than free epirubicin treatment in a colon cancer mouse model bearing C26 cells. Wang and colleagues (188) constructed nanolipodisks modified by glycosylated heptapeptide ATWLPPR (^9^G-A7R) containing 1-palmitoyl-2-oleoyl-sn-glycero-3-phosphocholine (POPC) and cholesterol plus 1,2-distearoyl-sn-glycero-3-phosphoethanolamine-N-[methoxy(polyethylene glycol)] (DSPE-PEG) to carry melittin and paclitaxel. ^9^G-A7R can specifically bind neuropilin-1 and vascular endothelial growth factor receptor 2 overexpressing in glioma cells, enhancing its proteolytic stability in serum and accessibility of A7R to the brain. The nanolipodisks described above could be prominently taken up by glioma. Paclitaxel combined with melittin protected against proteolysis and hemolysis, exhibited synergism against the human glioma cell line U87 and showed enhanced antiglioma properties in glioma-bearing mice. The glycopeptide-enabled paclitaxel/melittin delivery system showed significant anti-glioma effects in an intracranial glioma xenograft model (189). Photodynamic therapy (PDT) is to induce ROS production to induce immunogenic cell death for antitumor therapy. Liu and colleagues developed a serum albumin (SA)-coated boehmite (aluminum hydroxide oxide) organic-inorganic scaffold to envelop melittin and chlorin e6 (Ce6), a photosensitizer. Compared with free melittin treatment, this system significantly reduced hemolysis, enhanced intracellular ROS generation, and exhibited melittin-mediated enhancement of PDT effects, inhibiting tumor growth in a mouse model of breast cancer bearing 4T1 cells (176). Motiei and colleagues designed a polyelectrolyte nanocarrier via self-assembly of polyanionic and polycationic chains using folic acid-decorated polyethyleneimine as the shell, dextran sulfate as a complexing agent and polyglutamate grafted chitosan as the core (190). The nanocarrier above was used to deliver LTX-315, a 9-mer cationic amphiphilic peptide from bovine lactoferricin, melittin or miR-34a. LTX-315 can trigger an anticancer immune response, killing human cancer cells (191). miR-34a is a tumor suppressor miRNA. miR-34a downregulation is involved in the proliferation, invasion and metastasis of multiple types of cancers (192). The polyelectrolyte nanosystem carrying miR-34a, melittin or LTX-315 cold controls the release of genes and peptides and exhibits synergistic anti-tumor efficacy of melittin and miR-34a, such as arrest cell cycle and induce apoptosis of MDA-MB-231 cells (190). Asfour and colleagues designed a nanoconjugate of melittin and gabapentin and evaluated the synergistic effects against diabetic wounds (193). It was reported that gabapentin could promote wound healing (194). When conventional treatment fails, gabapentin can be used and compounded to promote dermal healing in the clinic (195). Under gabapentin-melittin-conjugated hydroxypropyl-methyl cellulose hydrogel treatment, wound contraction was expedited in streptozotocin-induced diabetic Wistar rats, suggesting potent wound healing activities. Furthermore, the gabapentin-melittin conjugate formulation increased the activity of antioxidant enzymes, reduced lipid peroxidation, inhibited the release of inflammatory factors, and enhanced proliferative and pro-collagen activities, indicating the synergistic effects of melittin with gabapentin on wound healing in diabetes. In addition, it was shown that a hydrogel formulation embedded tobramycin and melittin could efficiently alleviate biofilm-associated infections in a mouse wound model (196). Recently, Koide and colleagues engineered a temperature-responsive poly(N-isopropylacrylamide) (pNIPAm)-based copolymer. The nanosystem above could trap melittin at 37°C and swell and rapidly release melittin at 25°C. Furthermore, no hemolysis or cytotoxicity at 37°C occurred but hemolysis and cytotoxicity were observed at 25°C (33). Under tumor cooling treatment, melittin was released from the nanosystem only at the cooled site, and growth of tumor was inhibited in Colon26 NL-17-bearing mice *in vivo*, suggesting the applicable potential of melittin for anticancer therapy by local cooling-triggered site-specific release. Melittin modulation that have been reported in the literature are depicted in **Figure 2**.

## Conclusion

Melittin exhibits antiviral, antitumor, and anti-inflammatory functions, although there are practical challenges, such as hemolysis and drug safety. The scientific research on the functional analysis and mechanistic exploration of melittin has progressed, and the understanding of melittin has improved. Importantly, studies using nanomodification, immuno-conjugation, structural regulation and gene technology strategies provide insights into the potential applications of melittin in disease intervention and also for the exploration and application of similar antimicrobial peptides. At present, although there is no trial of melittin on humans that demonstrates the therapeutic properties and investigates the biosafety of melittin management in clinic, it is worth noting that increasing researches undoubtedly indicate the potential of melittin as a candidate for disease intervention. Due to the multi-target efficiency of melittin, further animal experiences should be performed to address the molecular mechanism of melittin action.

## Author contributions

HZ: Conceptualization, Investigation, Writing – review & editing. CS: Investigation, Writing – review & editing. NX: Conceptualization, Investigation, Writing – review & editing. WL: Investigation, Writing – review & editing.
