# Supplementary material for: The current landscape of the antimicrobial peptide melittin and its therapeutic potential
Source: Front Immunol. 2024 Jan 22;15:1326033. doi: 10.3389/fimmu.2024.1326033 (PMC10838977; doi:10.3389/fimmu.2024.1326033)
Supplement: Supplementary file 1 [file Table_1.docx]

**Supplementary Table S1 Antiviral activity of melittin against several kinds of virus**

| **Virus** | **Bioactivity of melittin** | **Molecular function** | **Model** | **Reference** |
| --- | --- | --- | --- | --- |
| HSV | Suppressed cell fusion mediated by HSV-1 syncytial mutants | Suppressed the activity of Na^+^ and K^+^ pump | Vero cells | 36 |
|  | Reduced viral supernatant titers, exhibited virucidal activity of HSV-1 | Directly interacted with the virus surfaces | Vero cells | 37 |
|  | Caused cell rounding and monolayer detachment, exhibited inhibitory effects on HSV-1 (strain F) and HSV-2 (strain G) | Affected ion gradients across the cell membrane | Vero cells | 31 |
| HIV-1 | Inhibited the virus replication by damaging HIV-1 proteins and mRNA synthesis | Interfered with host cell-directed gene expression of virus | KE37/1 cells | 52 |
|  | Captured more HIV-1 and significantly inhibit HIV-1 infections |  | VK2 vaginal epithelial cells | 53 |
| SARS-CoV-2 | Reduced the viral load | Affected metabolic and mRNA processing of the infected cells | Vero cells | 59 |
|  | Synergistic effects of melittin with Sitagliptin aganist SARS-CoV-2 | Molecular docking: melittin can bind to the RBD of SARS-CoV-2 spike protein | Vero E6 cells | 62 |
|  |  | Inhibited Mpro |  |  |
| Influenza A | Inhibited the replication of PR8/H1N1 | Exhibited direct effect on structure destabilization of the virus particle | Madin-Daby canine kidney cells | 37 |
|  | Reduced the viral titer in the lung of of H1N1-infected mice |  | H1N1-infected C57BL/6 mice |  |
| JV | Inhibited multiplication of JV |  | Vero cells | 64 |
| Enterovirus (EV-71) | Showed antiviral activity against EV-71 and ameliorated EV-71-mediated cytotoxic | Inhibited virus replication | Hela cells | 37 |
