# Supplementary material for: The current landscape of the antimicrobial peptide melittin and its therapeutic potential
Source: Front Immunol. 2024 Jan 22;15:1326033. doi: 10.3389/fimmu.2024.1326033 (PMC10838977; doi:10.3389/fimmu.2024.1326033)
Supplement: Supplementary file 2 [file Table_2.docx]

**Supplementary Table S2 Anti-tumor potentials of melittin in some kinds human cancers**

| **Cancer type** | **Properties of melittin** | **Molecular function** | **Modification on melittin** | **Model** | **Reference** |
| --- | --- | --- | --- | --- | --- |
| Colon cancer | Inhibited tumor growth |  | A temperature-responsive pNIPAm-based copolymer nanosystem with affinity for melittin at 37 ℃ and little affinity at 25 ℃ | Colon26 cells and NL-17-bearing mice | 33 |
|  |  |  | A calcium carbonate nanoparticles containing mucin 1–Dimer aptamers to carry epirubicin and melittin (mucin 1-Dimer aptamer-CCN nanosystem) | C26 cell | 34 |
|  | Tumoricidal effects on human colon carcinoma cells. Exerted synergistic effects with PLA2 against colon cancer cells | Disrupted the glycocalyx of the cell coat on the cell membrane.  Facilitated PLA2 directly act on the phospholipids of the lipid membrane bilayer |  | HCT116 cells | 70 |
|  | inhibitory effects on colon cancer cells in vitro | Arrested the carcinama cells in G2/M and pre-G1 phases | Fluvastatin nanoparticulate system composed with comprise phospholipid, alpha lipoic acid and melittin | Caco2 cells | 72 |
|  | Induced cancer cell death | Destroyed the cell membrane |  | COLO205 cells | 73 |
|  | Inhibited the growth, metastasis of cncer cells and increased inflammatory levels | Promoted the MHC-I cross-dressing by dendritic cells to prime tumor-specific CD8^+^ T cells | MnO2-melittin nanoparticles were conducted according to the situ redox reaction between poly(allylamine hydrochloride) and KMnO4 | MC38 cells | 74 |
| Gastric cancer |  | Rapid destroyed the cell membrane |  | AGS cells | 73 |
|  | Inhibits the growth of the human gastric cancer cells line.  Increased mitochondrial membrane permeability and reduced the mitochondrial membrane potential of cancer cells | Induced apoptosis of cancer cell through activating mitochondrial pathway |  | SGC-7901 cells | 77 |
| Colorectal cancer | Inhibitied cells growth of cancer cells | Caused cancer cells swelling and then shrink with moderated cell membrane damage |  | HCT-15 cells | 73 |
|  | Anti-tumorigenic effects against human colorectal cancer cells lines in vitro | Induced ER stress, leading to imbalance of calcium homeostasis.  Facilitated apoptosis |  | SW480 cells | 75 |
|  | Impeded the tumour growth | Triggered ER stress |  | SW480 tumor bearing mice | 76 |
|  | Inhibited the bone metastases in a human colorectal cancer mouse model | Caused necrosis and inflammation in the metastases |  | A mouse model of bone metastasis bearing HT-29 cells | 76 |
| Lung cancer | Impeded proliferation and caused apoptosis of cancer cells | Arrest cell cycle of cancer cells at the G1 phase |  | Chago-K1 cells | 80 |
|  | Inhibited proliferation and migration of cancer cells | Inhibited the differentiation of circulating monocytes into tumour-associated macrophages |  | THP-1 Cells, Wi-38 | 151 |
|  | Inhibit the growth of tumors in a mouse model of lung adenocarcinoma |  | A kind of lipid-coated polymer nanoparticles containing a lipid membrane intermediate layer, PFG and PEG targeting molecular shell based on intermolecular interactions of electrostatic attraction and hydrophobic effects | A nude mice bearing A549 cells | 169 |
| Breast cancer | Inhibit the tumor growth.  Inhibited lung metastasis in a TNBC breast cancer mouse model | Reduced M2-like tumor-associated macrophages | Conjugating fused melittin (melittin 8-26) and the pro-apoptotic peptide d(KLAKLAK)2 (dKLA) | 4T1 cells, mice bearing 4T1 TNBC cells | 82 |
|  | Inhibited the cells viability |  |  | SUM159 cells | 85 |
|  | Inhibitied cells growth of cancer cells.  Showed the synergies between melittin and docetaxel: enhancing the anti-tumor effects of docetaxel in the breast cancer mouse model | Reduced the protein expression of p-RTK and down-regulated PI3K/Akt signaling pathway.  Inhibited the activity of EGFR and HER2 | A bifunctional melittin peptide by adding an alpha-helical RGD peptide motif to N-terminal of melittin | T11 cell, T11 cells-bearing mice | 85 |
|  | Inhibited the viabiity of cancer cells |  | carbon nanoparticles, graphene oxide, pristine graphene, and diamond | MCF-7 and MDA-MB-231 | 86 |
|  | Tumoricidal effects | Increased the protein levels of urokinase-type plasminogen activator receptor (uPAR) and activated leukocyte-cell adhesion molecule (ALCAM), reduced the protein expressions of Lipocain-2 | Graphene-carried melittin | MDA-MB-231 | 87 |
|  | Inhibited the viabiity of cancer cells | Increased the protein expressions of deoxythymidine kinase, reduced the levels of ALCAM and The MHC class I chain-related gene A (MICA) |  | MCF-7 | 87 |
|  | Suppressed primary tumor growth |  | Hyaluronic acid and high-density lipoprotein were conjugated to envolope melitttin | 4T1 cells | 88 |
|  | Impede the growth of the metastatic sentinel lymph node |  |  | A mouse model with metastasis of breast cancer bearing 4T1 cell | 88 |
|  | Inhibited the migration of cancer cells |  |  | MDA-MB-231 cells | 90 |
|  | Induced apoptosis | Increased the expression of mitochondrial fusion protein 2 and dynamin-related protein 1 of mitochondria |  | 4T1 cells | 90 |
|  | Inhibits the growth of carcinoma cells | Suppressed the formation of TME by reducing the protein expression and mRNA levels of HIF-1α |  | MDA-MB-231 | 92 |
|  | Delayed the growth of subcutaneous murine tumors | Inducted the increases of ROS, triggering immunogenic cell death | A serum albumin (SA)-coated boehmite scaffold to load melittin and chlorin e6 (Ce6) | Breast cancer mouse model model of subcutaneous bearing 4T1 cells | 176 |
|  | Tumoricidal effects |  | An estrone-appended polyion complex micelle for carrying melittin |  | 179 |
|  | Exerted synergistic cytotoxicity with Epirubicin against tumor cells |  | mucin 1-Dimer aptamer-CCN nanosystem | MCF-7 cells | 34 |
|  | Showed synergistic anti-tumor effects with miR-34a | arrested cell cycle at G1/S phase and reduced the mRNA levels and protein expression of Cyclin D1 | A polyelectrolyte nanocarrier contains folic acid-modifided polyethyleneimine shell and polyglutamate grafted chitosan core. The shell is for delivering miR-34 and the core is for carryng melittin. | MDA-MB-231 cells | 190 |
| Hepatocellular carcinoma (HCC) | Inhibited the cell viability, migration and microfilament depolymerization of cancer cells.  Reduced tumor volume in the HCC mouse model. | Inhibited Rac1 |  | MHCC97L, MHCC97H cells and nude mice bearing MHCC97H cells | 32 |
|  | Inhibited the proliferation, migration and invasion of HCC cells | Regulated PI3K/AKT/mTOR signal pathway |  | MHCC97-H | 94 |
|  |  | Impeded the expression of DNMT1, regulated ADAMTS9-AS2 demethylation |  | HepG2 cells | 81 |
|  | Enhanced selectivity and anticancer effects of Sorafenib | Arrested the cell cycle of cancer cells at G2/M phase.  Increased intracellular ROS levels, reduced the potential of mitochondrial membrane and reduced the protein levels of p21 and XIAP |  | HepG2 cells | 94 |
|  | Impeded the proliferation, invasion and angiogenesis | Inhibited cathepsin S-induced angiogenesis.  Blocked the VEGF-A/VEGFR-2/MEK1/ERK1/2 pathway |  | MHCC97-H cells | 101 |
|  | Inhibited proliferation of tumor cells | Attenuatied the expression HDAC2 and upregulated PTEN |  | HepG2 cells | 108 |
|  | Suppressed the viability of tumor cells | Induced autophagy and activated mitochondrial apoptotic pathway |  | HepG2 cells | 114 |
|  | Specifically targeted and killed HepG2 cells |  | A homing peptide, SLSLITMLKISR (AM-2) was linked to melittin | HepG2 cells | 117 |
|  | Binded the surface of tumor cells and exhibited cytolytic activity | Anti-tumor selectivity targeting ASGPR-specific cytotoxicity | A recombinant immunotoxin through fusing an anti-ASGPR single-chain variable fragment to melittin | HepG2 cells | 118 |
|  | Inhibited the growth of the HCC cells.  Increased the overall survival of HCC mouse | Inhibited AFP-positive cancer cells in hypoxia microenviroment | An adenovirus vector encoding milittin gene under the control of HRE-AFP | Hep3B cells and Nude mice bearing Hep3B cells | 119 |
|  | Tumoricidal effects on HCC cells | Facilitated the death of cancer cells via inducing apoptosis | A non-viral vector containing survivin promoter to carry milittin gene | HepG2 cells, a mouse model bearing HepG2 cells | 120 |
|  | Inhibited the growth of cancer cells in vitro | Targeted AFP-positive HCC cells | A recombinant adenovirus vector with the AFP promoter encoding melittin gene by a bacterial homologous recombinant system | BEL-7402 cells | 121, 122 |
|  | Antineoplastic effects | Reduced PD-L1 protein levels and broke the bindings of PD-1 with PD-L1 | Wapping melittin with fluorinated EGCG. | HCC mouse model bearing Hep3B tumor cells | 185 |
| Prostate cancer | Inhibited cancer cell growth | Inducted aoptotic cell death in tumor cells via suppressing NF-κB and activiting Caspase signal |  | LNCaP, DU145, and PC-3 cells | 123 |
|  | Reduced cell viability of cancer cells | Specifically target to bind matrix MMP2 and inhibit the enzymatic activity of MMP2 | A chlorotoxin-targeted nanovector for delivering melittin gene | PC-3 cells | 126 |
|  | Inhibit the proliferation and migration of cancer cells (CRPC)  and enhanced cisplatin sensitivity | down-regulated LCN2 |  | PC-3 cells | 127 |
| Melanoma | Significantly caused the decline in the survival rate of tumor cells |  | Hyaluronic acid-modified liposomes to carry melittin | B16F10 | 170 |
|  |  |  |  | B16 | 74 |
|  | Inhibited tumor growth |  | A hybrid cytolytic peptide by linking α-helical peptide to the N-terminus of melittin via a GSG linker (α-melittin) | C57BL/6 mice subcutaneously implanted with B16F10 cells | 181 |
|  | Impede the primary and distant tumor growth |  | α-melittin | Melanoma mouse model caused by intradermally implanted with B16F10 cells | 182 |
| Glioma | Showed synergistic effects with paclitaxel against glioma cells and prolonged the survival time of glioma-bearing mice |  | 9G-A7R-modified nanolipodisks to melittin and paclitaxel | U87 cells and BALB/c nude mice intracranially inoculated U87 cells | 189 |
