# Supplementary material for: The current landscape of the antimicrobial peptide melittin and its therapeutic potential
Source: Front Immunol. 2024 Jan 22;15:1326033. doi: 10.3389/fimmu.2024.1326033 (PMC10838977; doi:10.3389/fimmu.2024.1326033)
Supplement: Supplementary file 3 [file Table_3.docx]

**Supplementary Table S3 Anti-inflammatory effects of melittin in several kinds of human inflammation-related disease**

| **Stimuli/Models/Human samples** | **Bioactivity** | **Molecular target** | **Strain/Species** | **Potential for treating inflammation-associated disease** | **Reference** |
| --- | --- | --- | --- | --- | --- |
| LPS | Suppressed microglial proinflammatory responses | Inhibited LPS-primed inflammatory response through suppressing the activation of NF-κB via the regulation of the IκBα signaling pathway.  Attenuated NO production via the inhibition of the JNK and Akt pathway. | BV2 cells | Neurodegenerative diseases accompaning by the activation of microglia | 128 |
| H_2_O_2_ | Protect against H_2_O_2_-induced apoptosis | Mitigated H_2_O_2_-induced decreases in the mRNA levels and protein expression of Bcl-2 and suppressed H_2_O_2_-triggered increases in the mRNA and protein expression of Caspase-3 | SH-SY5Y | Neurodegenerative diseases | 130 |
| A mouse model for inherited ALS | Improved motor function and reduced death of neuronal cells in the spinal cord | Alleviated microglial overactivation, reduced the level of phosphorylated p38 and the α-synuclein misfolding and restored the proteasomal activity in the brainstem and spinal cord | hSOD1G93A mouse | ALS-related neuroinflammation | 131 |
| LPS | Inhibited MMP3 production | Suppressed LPS-induced increases in the DNA-binding activity of NF-κB. | Fibroblast-like synoviocytes | Rheumatoid Arthritis | 133 |
| Patients with Rheumatoid Arthritis | Suppressed the cells viability and inhibited IL-1β secretion | Promote apoptosis and autophagy | Fibroblast-like synoviocytes | Rheumatoid Arthritis | 134 |
| Sodium nitroprusside, TNF-α and LPS | Melittin exhibited high affinity interactions with IKKα and IKKβ | Blockade inflammatory stimulus-induced IkB release and NF-kB activation | Raw 264.7 mouse macrophages | Rheumatoid Arthritis | 141 |
| Patients with Rheumatoid Arthritis | Reduced the levels of inflammatory mediators, NO and PGE2.  Suppressed the expressions of iNOS and COX-2 |  | Fibroblast-like synoviocytes |  |  |
| LPS and sodium nitroprusside | Reduced inflammatory stimuli-induced NO and PGE2 production | Inhibited the activity of NF-κB via JNK signaling pathway | Raw 264.7 cells, THP-1 human monocytic cells and Fibroblast-like synoviocytes from Rheumatoid Arthritis patients | Rheumatoid Arthritis | 143 |
| Acetic acid-induced Ulcerative colitis (UC) through rectal perfusion | Protected mucosa and submucosa of colon by performing anti-inflammatory and anti-oxidative effects in the UC mouse model | Inhibited NF-κB and p38 MAPK via downregulating TLR4/TRAF6 | Swiss albino mice | UC | 145 |
| Dextran sulfate sodium (DSS)-induced colitis models | Alleviated the mucosal damages induced by UC |  | C57BL/6 mice | UC | 146 |
|  | Exhibited synertic effects with Ulfasalazine against UC | Ameliorated the imbalance of redox triggered by UC |  |  |  |
| D-galactosamine/LPS-  induced acute liver failure | Reduced the mortality rate of acute liver failure mice | Suppressed the upregulation of NF-κB DNA-binding activity in the acute liver failure mice | C57BL/6 mice | Acute hepatic failure | 149 |
|  | Alleviated hepatocellular damage, mitigated the area and extent of necrosis and reduced the infiltration of inflammatory cells |  |  |  |  |
| LPS | alleviated inflammation | Suppressed PKM2 and disrupted the Warburg effect |  | RAW264.7 macrophages | 150 |
| D-galactosamine/LPS-  induced acute liver failure | mitigated hepatic inflammation and reduced mortality rate of Acute liver failure mice | Inhibited the aerobic glycolysis by impeding PKM2/HIF-1α | C57BL/6 mice | Acute liver failure |  |
| Heat-killed Propionibacterium acnes (P. acnes) | Increased the viability, suppressed P. acnes-triggered apoptosis and arresting of THP-1 cells in the sub-G1 phase | Reduced the cleavage of caspase-3, caspase-8 and PARP | THP-1 monocytic cells | Acne | 151 |
| Heat-killed P. acnes | Suppressed P. acnes-triggered flammatory cytokines secretion | Reduced the expression of TNF-α and IL-1β through regulating NF-kB and MAPK signaling pathways | HaCaT cells | Inflammatory skin diseases | 152 |
| *P. acnes*-induced inflammatory skin lesions | Showed anti-inflammatory effects against the inflammation-related skin lesions caused by living P. acnes | Suppressed NF-κB and AP-1 | ICR mice |  |  |
| Ovalbumin (OVA) | Suppressed OVA-induced production of Th2 cytokines and TSLP | Prevented from filaggrin deficiency and STAT3 activation via the decreases of IL-4 and IL-13 | HaCaT cells | Atopic dermatitis | 155 |
|  | Ameliorated OVA-induced skin thickening and inflammatory infiltration | Reduced the exaggerated IgE response | BALB/c mice |  |  |
| Porphyromonas Gingivalis LPS (PgLPS) | Exerted protective effects against PgLPS-mediated injury in the keratinocytes | Melittin suppressed PgLPS-mediated increases of proinflammatory cytokines by suppressing NF-κB, ERK and Akt signaling | HaCaT | Periodontitis | 157 |
